# Supplementary material for: From sequence to enzyme mechanism using multi-label machine learning
Source: BMC Bioinformatics. 2014 May 19;15:150. doi: 10.1186/1471-2105-15-150 (PMC4229970; doi:10.1186/1471-2105-15-150)
Supplement: Additional file 2 — Java code of ml2db. Additional file ml2db_code.tar.gz contains the Java source code to run the multi-label machine learning experiments and save the results to database. The code’s Javadoc is included. [file 1471-2105-15-150-S2.zip › additional file 2/ml2db/ml2db/doc/test/ml2db/al/utility/globaldistance/DistanceExperimentDetailsTest.html]

DistanceExperimentDetailsTest


JavaScript is disabled on your browser.


- Overview
- Package
- Class
- Use
- Tree
- Deprecated
- Index
- Help

- Prev Class
- Next Class

- Frames
- No Frames

- All Classes

- Summary:
- Nested |
- Field |
- Constr |
- Method

- Detail:
- Field |
- Constr |
- Method


test.ml2db.al.utility.globaldistance

## Class DistanceExperimentDetailsTest

- java.lang.Object
- - test.ml2db.al.utility.globaldistance.DistanceExperimentDetailsTest

- ---

    

  ```
  public class DistanceExperimentDetailsTest
  extends java.lang.Object
  ```

  Class

  Version:
  :   8 Jul 2011

  Author:
  :   Luna De Ferrari ldf(at)st-andrews.ac.uk

- - ### Constructor Summary

    Constructors

    Constructor and Description

    `DistanceExperimentDetailsTest()`
  - ### Method Summary

    Methods

    | Modifier and Type | Method and Description |
    | --- | --- |
    | `static ExperimentDetails` | `getExperimentManagerTestsetWithPositioning()` |
    | `static ExperimentDetails` | `getExperimentManagerTrainsetWithPositioning()` |
    | `static ExperimentDetails` | `getExpManTestsetWithGlobalPositAndConfidence()` |

    - ### Methods inherited from class java.lang.Object

      `equals, getClass, hashCode, notify, notifyAll, toString, wait, wait, wait`

- - ### Constructor Detail


    - #### DistanceExperimentDetailsTest

      ```
      public DistanceExperimentDetailsTest()
      ```
  - ### Method Detail


    - #### getExperimentManagerTestsetWithPositioning

      ```
      public static ExperimentDetails getExperimentManagerTestsetWithPositioning()
      ```


    - #### getExperimentManagerTrainsetWithPositioning

      ```
      public static ExperimentDetails getExperimentManagerTrainsetWithPositioning()
      ```


    - #### getExpManTestsetWithGlobalPositAndConfidence

      ```
      public static ExperimentDetails getExpManTestsetWithGlobalPositAndConfidence()
      ```


- Overview
- Package
- Class
- Use
- Tree
- Deprecated
- Index
- Help

- Prev Class
- Next Class

- Frames
- No Frames

- All Classes

- Summary:
- Nested |
- Field |
- Constr |
- Method

- Detail:
- Field |
- Constr |
- Method
